# Myocardial, Valvular, and Vascular Structural and Functional Properties in Acromegaly

**DOI:** 10.3390/jcm12216857

**Published:** 2023-10-30

**Authors:** Attila Nemes, Árpád Kormányos, Nóra Ambrus, Csaba Lengyel, Zsuzsanna Valkusz

**Affiliations:** Department of Medicine, Albert Szent-Györgyi Medical School, University of Szeged, 6725 Szeged, Hungary; kormanyos.arpad@med.u-szeged.hu (Á.K.); ambrus.nora@med.u-szeged.hu (N.A.); lengyel.csaba@med.u-szeged.hu (C.L.); valkusz.zsuzsanna@med.u-szeged.hu (Z.V.)

**Keywords:** acromegaly, cardiac mechanics, vascular, remodeling

## Abstract

Acromegaly is an uncommon systematic endocrine disease caused by the hypersecretion of human growth hormone and, consequently, of insulin-like growth factor-1 during adulthood. Acromegaly could cause a typical cardiomyopathy characterized by left ventricular hypertrophy associated with diastolic dysfunction, which later could progress to systolic dysfunction. Moreover, some valvular and vascular abnormalities are also associated with acromegaly. This present review aims to summarize available information regarding acromegaly-associated abnormalities in myocardial, valvular, and vascular structural and functional properties and their relationship to disease activity and treatment options.

## 1. Acromegaly and Cardiovascular Abnormalities

Acromegaly is an uncommon systematic endocrine disease caused by the hypersecretion of human growth hormone (hGH) and, consequently, of insulin-like growth factor-1 (IGF-1) during adulthood with physical changes occurring slowly over several years [1,2]. Untreated acromegaly is associated with increased morbidity and mortality due to significant complications such as increased risk of type 2 diabetes mellitus (DM), metabolic, gastrointestinal, and neurological abnormalities, tumors, cardiovascular alterations, etc. The latter is one of the most important abnormalities affecting the quality of life due to the fact that acromegaly could progress into a typical cardiomyopathy in the final stage of the disease [3]. The first changes seen during the course of the disease are hyperkinetic syndrome and left ventricular (LV) hypertrophy, followed by a more prominent LV hypertrophy with impaired diastolic ventricular filling caused by increased calcium sensitivity and, consequently, increased myocardial contractility. In more advanced stages, extracellular collagen deposition is seen, leading to reduced systolic and diastolic LV function, remodeling of the LV, and heart failure [4,5,6,7].

## 2. Acromegaly and Cardiovascular Imaging

In the past decades, cardiovascular imaging has undergone significant development. Not only have new procedures such as computer tomography and magnetic resonance imaging spread in the routine practice, but echocardiography has developed significantly as well. Due to this development, more detailed volumetric and functional chamber quantifications and the assessment of valvular and vascular functions are available now. Moreover, a complete analysis of myocardial mechanics can now be performed. One of the latest developments is three-dimensional (3D) speckle-tracking echocardiography (STE), which is a relatively new non-invasive echocardiographic tool with the capability to see the chambers in 3D, allowing not only accurate volumetric measurements respecting the cardiac cycle but an assessment of the deformation (strain) and rotational features of cardiac chambers at the same time using the same acquired 3D echocardiographic datasets using virtual 3D models [8,9,10,11]. Since 2011, a clinical study has been organized at the Cardiology Department of the University of Szeged, Szeged, Hungary, to assess the clinical usefulness and prognostic impact of three-dimensional speckle-tracking echocardiography-derived parameters in certain pathologies, including acromegaly. This study is called “Motion Analysis of the heart and Great vessels bY three-dimensionAl speckle-tRacking echocardiography in Pathological cases” (MAGYAR-Path) Study.

The aim of this publication was to review item by item the findings related to acromegaly in myocardial, valvular, and vascular structural and functional properties, including those first described in the MAGYAR-Path Study [12,13,14,15,16,17,18,19]. Regarding vascular properties, only aortic and pulmonary artery-related abnormalities were detailed. The results of publications were summarized from the past decades when the majority of the patients were diagnosed in time and treated accordingly. The findings should be interpreted in this light.

## 3. The Left Heart and the Aorta

### 3.1. Left Ventricle

#### 3.1.1. Under Healthy Circumstances

**Figure 1:**
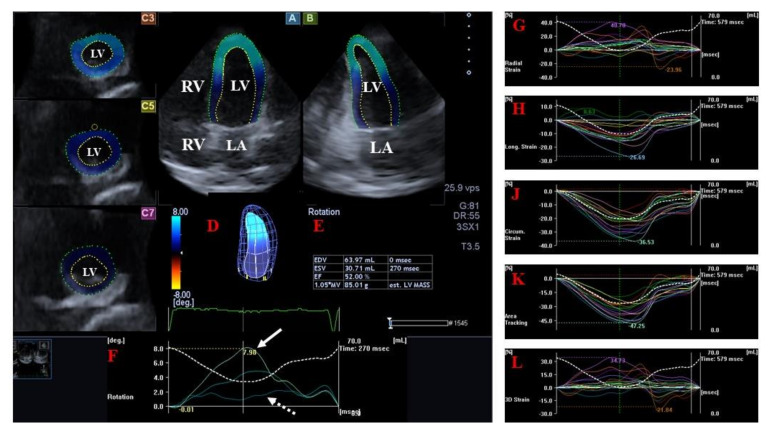
Three-dimensional (3D) speckle-tracking echocardiographic assessment of the left ventricle. From the acquired 3D echocardiographic dataset, using dedicated vendor-provided software, the following views are created automatically during the assessment of the LV: longitudinal apical 4-chamber (**A**) and two-chamber (**B**) views and short-axis views at the apical (**C3**) and midventricular (**C5**) and basal (**C7**) LV regions. The 3D cast of LV (**D**), calculated LV volumetric data (**E**), and apical (white arrow) and basal (white dashed arrow) LV rotations (**F**) together with time–LV global (white curve) and segmental (colored curves) radial (**G**), longitudinal (**H**), circumferential (**J**), area (**K**), and 3D (**L**) strain curves with time–LV volume changes curve (dashed white curve) are presented. In the image, an acromegaly patient with LV “rigid body rotation” is presented, which means that both apical and basal LV rotations have the same counterclockwise-oriented direction, leading to the absence of LV twist. Abbreviations. LV = left ventricle, LA = left atrium, RV = right ventricle, EDV = end-diastolic volume, ESV = end-systolic volume, EF = ejection fraction, MASS = LV muscle mass.

In the bullet-shaped LV, subepicardial fibers run in a left-handed direction, fibers in the mid-layer run circumferentially, while subendocardial fibers run in a right-handed direction [20]. In healthy subjects, during systole, the LV stroke volume is ejected through the semilunar aortic valve towards the ascending aorta with the mitral valve closed. In diastole, the LV fills with blood from the left atrium (LA) through the mitral valve with the aortic valve closed. The walls of the LV have a special 3D contractility pattern with radial (thickening/thinning), longitudinal (lengthening/shortening), and circumferential (widening/narrowing) contraction/relaxation represented via echocardiographic unidimensional/unidirectional (LV-RS, LV-LS, and LV-CS, respectively) or combined multidimensional/multidirectional strains (LV area strain, LV-AS and LV 3D strain, LV-3DS) [8,9,10,11]. Moreover, although these spatial movements cannot be separated from each other, the basal regions of the LV rotate clockwise in systole, while the apex of the LV moves in opposite direction and has a counterclockwise rotation in a healthy heart. Given that the apex and the base of the LV move in opposite directions, a twisting movement is created, which is similar to wringing a towel. Therefore, this movement is called an LV twist. In diastole, the movement back to baseline state is called LV untwisting [20,21] (Figure 1).

#### 3.1.2. In Acromegaly

##### LV Structure, Volumes and Hypertrophy

In a study assessing cats with acromegaly, 7 out of 11 subjects had LV concentric hypertrophy and showed signs of LV diastolic dysfunction [22]. Early studies conducted over 20 years have confirmed significant LV abnormalities in acromegaly [23,24,25]. In a human study evaluating three acromegaly patients with heart failure, dilated LV with diffuse hypokinesis and LV systolic dysfunction without LV hypertrophy were found [23]. In another paper, various degrees of LV hypertrophy could be demonstrated in 11 out of 25 patients. LV was dilated in 6 out of 25 patients, which was associated with LV systolic dysfunction in most cases [24]. LV hypertrophy and hGH level seemed to be associated [25,26].

Patients who develop hGH deficiency following the cure of acromegaly did not demonstrate elevated LV mass, in contrast to patients with a history of acromegaly but normal hGH levels or patients with active acromegaly [26]. Using cardiac magnetic resonance imaging, 72% of patients with untreated active acromegaly had LV hypertrophy, which was only detected in 36% of patients via echocardiography at the time of the examinations. However, cardiac fibrosis was absent in all patients, irrespective of the estimated duration of the disease [27]. In a recent study, the prevalence of LV hypertrophy in acromegaly patients was 67% (78% concentric, 22% eccentric) [28]. 

Diastolic dysfunction is a frequent finding in acromegaly, which could be detected with different kinds of echocardiographic methods [29]. LV diastolic dysfunction was present in 11.3% of the patients with higher LV mass in acromegaly patients [30]. The risk of LV diastolic dysfunction seems to be related to the presence of diabetes mellitus (DM) and advanced age and is independent of disease duration and activity [31]. hGH positively correlated with the Tei index, representing LV performance and LV diastolic dysfunction [32]. In an early study, an improvement of LV diastolic function and a significant improvement of LV hypertrophy in patients with inactive acromegaly and normal systolic cardiac function could be detected compared to those with active disease [33].

Some of the cardiac involvements in acromegaly patients were reversible 12 months after transsphenoidal hypophysectomy with the consequent lowering of hormone levels, including reversed LV hypertrophy and recovered LV systolic dysfunction [34]. In another study, following successful transsphenoidal surgery for acromegaly, significant improvements in LV mass index and diastolic filling were found 6 months post-surgery [35]. Similarly, after surgical excision of pituitary macroadenoma, significantly improved LV mass and LV ejection fraction were found in cured patients and those with glucose-suppressed hGH concentrations between 1 and 5 µg/L [36]. LV mass index regressed in men but not in women following lanreotide Autogel therapy [37]. The beneficial effects of somatostatin analog treatment have been confirmed in an early meta-analysis [38]. Significant reductions in the LV mass index and the mean wall thickness/internal end-diastolic radius ratio, as well as improvements in diastolic filling parameters, could be detected, following 6 months of somatostatin analog octreotide treatment [39]. There were significant improvements in LV hypertrophy and diastolic and systolic dysfunction following 5 years of depot somatostatin analogs as first-line therapy in acromegaly [40]. Moreover, with long-term combined treatment with somatostatin analogs and pegvisomant, improvements in LV mass index and diastolic dysfunction were seen in patients who were resistant to high-dose somatostatin analog monotherapy [41]. Similarly, improved LV hypertrophy and diastolic filling were found 12 months after first-line treatment with somatostatin analog or surgery [42]. Following 18 months of pegvisomant therapy, not only reduced prevalence of LV hypertrophy but improved systolic and diastolic function could be demonstrated in active acromegaly patients [43]. In contrast, no differences in echocardiographic parameters between patients with controlled and uncontrolled acromegaly could be detected, suggesting that the structural and functional changes did not reverse with biochemical control in another study [30]. In patients with acromegaly who had undergone transsphenoidal adenomectomy, the cardiac structure did not differ among the groups with different levels of hGH control [44]. Moreover, first-generation somatostatin analogs had a beneficial effect on LV mass index, however, no firm conclusion could be reached for other parameters (LV ejection fraction (EF), LV diameter, E/A ratio) [45].

##### LV Strains and Rotational Parameters

In an earlier two-dimensional STE study, no impairment of LV strain could be demonstrated in acromegaly [46]. In another study, higher LV mass and impaired systolic function (lower LV-EF and LV-GLS) could be detected [7]. However, these abnormalities represented subclinical impairment due to the fact that LV-EF was still above the normal cut-off value. Same results were found in treatment-naive acromegaly patients, presenting abnormal LV-GLS, indicating subclinical systolic dysfunction. Hypertension and DM were not found to be significant determinants of abnormal LV-GLS [47]. However, the presence of DM in acromegaly resulted in the further deterioration of LV deformation represented by the reduction in LV-RS from the MAGYAR-Path study [16]. In a color Doppler myocardial imaging study in acromegaly patients with active untreated disease, impaired diastolic function could be detected with an impairment of regional myocardial systolic function, which improved with medical treatment of acromegaly [48]. In 140 patients with acromegaly, increased interventricular septum and LV posterior wall and LV mass index could be detected, which was accompanied by reduced LV diastolic function with preserved LV systolic function represented by normal LV-EF. However, global LV-LS was slightly lower [6]. Global LV-LS was found to be reduced in 48% of patients with active acromegaly and systolic blood pressure (BP), N-terminal (NT) prohormone of brain natriuretic peptid (BNP), IGF-1, LA diastolic diameter, LV mass index were associated with global LV-LS [49]. In another study, acromegaly patients (83% having the active disease) showed structural changes: a higher LV-indexed mass and relative wall thickness were detected compared to controls. Acromegaly patients had functional changes, including reduced LV-EF, altered LV diastolic function and impaired LV-LS and LV-RS, compared to controls [50]. In another study, despite the higher prevalence of LV hypertrophy, patients with long-term acromegaly had similar LV-EF, global LV-LS, and LV-CS compared to controls [51]. In contrast, active acromegaly was found to be associated with enhanced LV-RS and preserved LV-LS from the MAGYAR-Path Study. Only LV-CS differed between active and inactive acromegaly patients [19]. In a recent study, the effect of acromegaly treatment on LV systolic function was visible even after 3 months of preoperative somatostatin receptor ligand treatment, especially in women. Patients with surgical remission had better global LV-LS compared to patients with persistent acromegaly [52]. In a comparative combined 3DSTE study from the MAGYAR-Sport and MAGYAR-Path studies, both elite athletes playing high-dynamic sports and acromegaly patients had dilated LV and LV deformation abnormalities with differences in the nature and extent of these alterations compared to healthy subjects not exercising regularly [17].

The first findings, in which LV rotational mechanics were examined, impaired 3DSTE-derived LV apical rotation and twist could be demonstrated from the MAGYAR-Path Study. The near absence of LV twist, called LV “rigid body rotation” (LV-RBR), was found in 20% of acromegaly cases [15,18]. Diabetic acromegaly patients had tendentiously higher LV apical rotation and twist with a similar ratio of LV-RBR compared to non-diabetic cases [16]. A comparison of LV rotational mechanics showed dissimilarities between acromegaly patients and elite athletes playing high-dynamic sports [17]. In contrast, LV twist did not differ between active acromegaly patients with no detectable heart disease and controls in another study [51].

### 3.2. Left Atrium

#### 3.2.1. Under Healthy Circumstances

**Figure 2:**
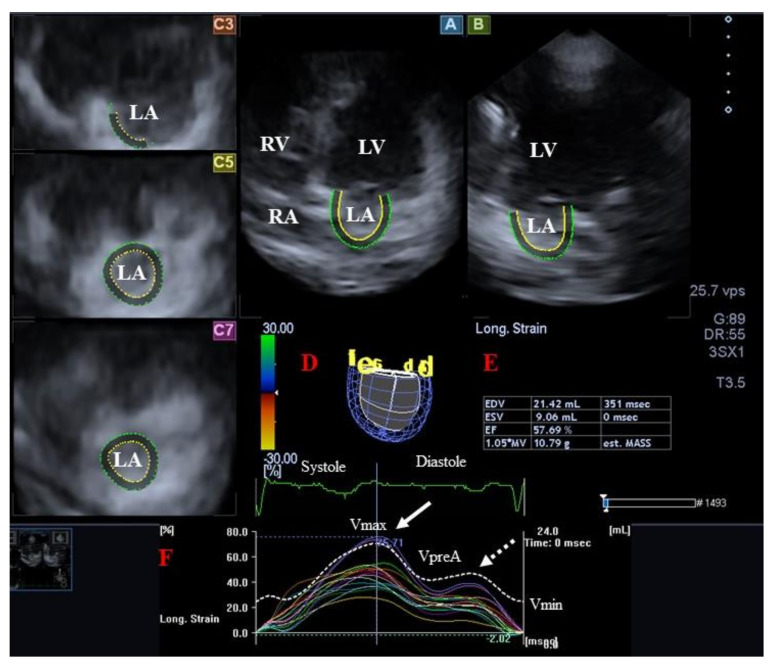
Three-dimensional (3D) speckle-tracking echocardiographic assessment of the left atrium (LA). From the acquired 3D echocardiographic dataset, the following views are created automatically: longitudinal apical four-chamber (**A**) and two-chamber (**B**) views are presented together with short-axis views at the basal (**C3**), midatrial (**C5**), and superior (**C7**) LA regions. Virtual 3D model of the LA (**D**), calculated LA volumetric data (**E**) and time–LA global (white curve) and segmental (colored curves) longitudinal (**F**) strain curves with time–LA volume changes curve (dashed white curve) are also presented. White arrow represents peak LA strain, while dotted white arrow represents LA strain at atrial contraction. Abbreviations. LV = left ventricle, LA = left atrium, RV = right ventricle, RA = right atrium, EDV = end-diastolic volume, ESV = end-systolic volume, EF = ejection fraction, MASS = LA muscle mass, Vmax = minimum end-systolic LA volume, VpreA = early diastolic LA volume before atrial contraction, Vmin = end-diastolic minimum LA volume.

LA is located on the left posterior side of the heart, and its walls consist of circumferential and longitudinal muscle bundles. The circumferential ones (e.g., interatrial band) are located at the base of the LA, while the longitudinal ones (e.g., septoatrial band) predominate in the parietal walls [53,54]. The rim of the oval fossa is the most important muscular structure of the septal surface, formed by the folded atrial walls, to which the other main muscles of the atrium are connected, providing mechanical support for the movement of the atrial walls. The LA wall is thin but thicker than that of the right atrium (RA) [54]. From a physiologic aspect of view, LA has a distinct phasic function during the cardiac cycle, being a reservoir in LV systole, a conduit in early LV diastole, and acting as a booster pump in late LV diastole [55]. LA volume is maximal in end-systole, having a pre-atrial contraction volume in early diastole and minimal in late diastole. From LA volumes, several stroke volumes (SVs) and emptying fractions (EmFs) featuring all phases of the LA function can be calculated. Novel echocardiographic methods allow for a more detailed assessment of LA [56,57] (Figure 2).

#### 3.2.2. In Acromegaly

The number of studies in which LA was examined in acromegaly is limited. In an animal study, 6 out of 11 cats with acromegaly showed enlarged LA [22]. Human studies confirmed the dilation of LA in acromegaly patients with and without heart failure [1,6,7,23,28,30]. LA volume index was higher in acromegaly patients [30]. With advances in cardiovascular imaging techniques, more detailed analysis could be performed. Both inter- and LA intra-atrial electro-mechanical delay (AEMD) were prolonged in patients with acromegaly. More prolonged intra-AEMD values were seen in patients with active acromegaly than in those with inactive acromegaly. IGF-1 was an independent predictor of inter-AEMD in patients with acromegaly [58]. In a recent study, greater LA anteroposterior diameter, indexed LA volume, and impaired echocardiographic strain parameters corresponding with LA function could be detected by STE [7]. It is in agreement with other findings demonstrating reduced LA strain in active acromegaly, which was associated with systolic BP, NT-BNP, IGF-1, LA diastolic diameter, LV mass index, and global LV-LS [49]. The reversibility of abnormalities is an important question: 12 months after transsphenoidal hypophysectomy, LA showed signs of remodeling [34].

In a recent detailed analysis from the MAGYAR-Path Study, LA volumes representing all phases of LA function were found to be increased in acromegaly. Moreover, these abnormalities were present regardless of the disease activity. From LA functional properties, systolic LA total atrial SV and late diastolic LA active atrial SV were increased without changes in early diastolic LA passive atrial SV and all LA-EmFs. Most LA volumes and volume-based functional properties were increased in inactive acromegaly patients, suggesting that preexisting LA volume changes do not resolve after proper treatment, and enhanced LA-SVs might suggest a compensatory mechanism. 3DSTE is not only suitable for measuring the volumetric and volume-based functional properties of LA at the same time. As with LV, LS, CS, and RS, as well as combined strains, can be measured, the clinical suitability of which requires further investigations. Strain analysis revealed decreased LA-CS and increased LA-RS and LA-3DS in acromegaly in the systolic reservoir phase. In contrast with volumetric data, in inactive acromegaly, decreased LA-CS receded [13]. However, these results conflict with recent findings demonstrating no differences in LA volumes and mechanical functions between active acromegaly patients and matched controls without correlations with hGH and IGF-1 levels [59].

### 3.3. Mitral Valve

#### 3.3.1. Under Healthy Circumstances

**Figure 3:**
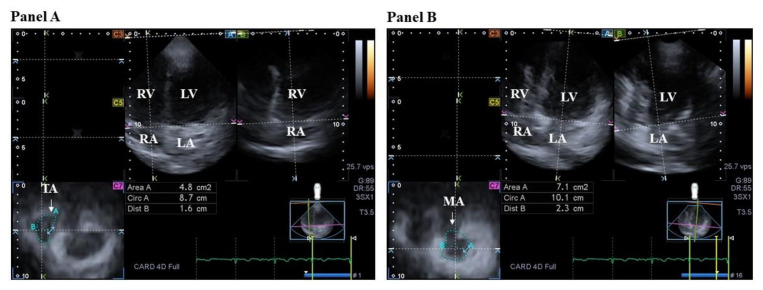
Three-dimensional (3D) speckle-tracking echocardiographic presentation of the two-dimensionally projected views of tricuspid (TA) (**panel A**) and mitral annuli (MA) (**panel B**). From the acquired 3D echocardiographic dataset, the following views can be created: apical four-chamber (A) and two-chamber views (B) and a cross-sectional view at the level of the TA/MA optimized in apical four- and two-chamber views (C7). The white arrow represents the TA/MA plane. Abbreviations: LA = left atrium, LV = left ventricle, RA = right atrium, RV = right ventricle, Area = TA/MA area, Circ = TA/MA perimeter, Dist = TA/MA diameter.

The 3D saddle-shaped mitral valve (MV) has a dynamic motion during the cardiac cycle [60]. MV involves a fibrous mitral annulus (MA), anterior and posterior leaflets, tendineal chords, and papillary muscles. While MV is open with a normal one-way unidirectional flow from LA to LV in diastole, MV is closed without backflow (mitral regurgitation, MR) due to primary or secondary reasons in systole. Under healthy circumstances, myocardial contraction of adjacent LA and LV areas respecting the cardiac cycle and occurring at the appropriate time is required for proper MA motion [61,62] (Figure 3).

#### 3.3.2. In Acromegaly

Mitral valve abnormalities (annular and/or leaflet fibrosis, thickening, calcification, and regurgitation (MR)) were present in 64% and 55% of active and cured acromegaly patients [63]. In another study, the prevalence of MR (≥moderate severity) was present in 5% of acromegaly patients. The prevalence of valvular regurgitation in acromegaly patients significantly correlated to the duration of the disease. Cases with or without active disease were found to have similar prevalence rates. Moreover, acromegaly-related valvular damage seemed irreversible in contrast to LV hypertrophy [64]. This result is in accordance with a recent finding demonstrating MA dilation in acromegaly regardless of its activity without MA functional impairment from the MAGYAR-Path Study [12]. Preserved MA functional properties calculated on the basis of the MA dimensions could be explained by the increased function of adjacent LA and LV areas (see above). 

### 3.4. Aortic Valve

#### 3.4.1. Under Healthy Circumstances

The semilunar aortic valve is located between the aorta and the outflow tract of the LV, having three thin leaflets ensuring one-way blood flow towards the systemic circulation [65].

#### 3.4.2. In Acromegaly

Aortic valve abnormalities (fibrosis, thickening, calcification, ectasia, and mild to moderate regurgitation) were present in 48% and 59% of active and cured acromegaly patients [63]. In another study, aortic valve regurgitation (>/≥trace severity) was increased in acromegaly with a prevalence of 30% of patients, which was dependent on the duration of exposure to increased hGH [64].

### 3.5. Aorta

#### 3.5.1. Under Healthy Circumstances

The aorta is the largest artery, which significantly interacts with the LV and LA [66]. The aorta is not a rigid tube but has a strong impact on regulating blood flow via the Windkessel effect due to its elasticity [67]. The stiffening of the aorta may contribute to LV abnormalities by increasing systolic BP, decreasing diastolic BP, and thus increasing LV afterload, which could promote LV hypertrophy, impair LV relaxation, leading to LV diastolic dysfunction, and compromise coronary perfusion [67].

#### 3.5.2. In Acromegaly

There are confusing findings regarding aortic abnormalities in acromegaly. Dimensions of the aortic root and the ascending aorta (AA) were found to be larger in acromegaly patients than in healthy people [6,68,69]. Moreover, expanding AA mainly depended on the duration of the disease instead of serum hGH and IGF-1 level [68]. 

Several methods have been known for a long time that are available for the non-invasive determination of aortic stiffness [70]. There is a strong agreement in the literature that aortic stiffness is increased in acromegaly regardless of the method of assessment, featured by echocardiographic aortic elastic properties [71,72], central arterial pressure waveforms derived from measured radial artery waveforms [73], pulse wave velocity (PWV) [30,74,75,76,77,78], or cardio-ankle vascular index (CAVI) [79]. Increased aortic stiffness could be demonstrated as independent of BP elevation as well [80]. Active acromegaly was associated with lower CAVI compared to healthy cases, and CAVI was negatively correlated with the serum IGF-I level [79]. While the echocardiographic aortic stiffness index (ASI) was similarly higher, the aortic strain and distensibility were decreased in patients with acromegaly being in remission and active acromegaly compared to controls without differences between these acromegaly groups [72]. Arterial stiffness was increased in patients with acromegaly with cardiovascular risk factors, and that increased arterial stiffness was associated with hemodynamic (systolic and diastolic BPs) and metabolic (body mass index, fasting plasma glucose level, and HbA1C level) parameters [81]. PWV was decreased in acromegaly patients compared to non-acromegalic control participants with similar cardiovascular risk profiles [82]. In patients with active disease, preclinical markers of atherosclerosis were found to be worse compared to patients with inactive disease, but the role of diabetes and hypertension prevailed in hGH excess [78]. It was confirmed when facilitated aortic stiffness was found in diabetic acromegaly patients compared to non-diabetic ones [83]. Moreover, the prognostic significance of echocardiographic ASI could also be demonstrated [84]. 

Despite successful surgery, acromegaly patients showed significantly decreased arterial distensibility [85]. In patients with acromegaly who had undergone transsphenoidal adenomectomy, vascular stiffness did not differ among the groups with different levels of hGH control [44]. However, improvement in PWV could be demonstrated following lanreotide Autogel therapy [37]. No differences in PWV between patients with controlled and uncontrolled acromegaly could be detected, suggesting that the structural and functional changes do not reverse with biochemical control [30].

In addition to the altered function of the aorta, vascular endothelial function may also have abnormalities affecting both flow-mediated dilation and endothelial cell markers in acromegaly [77,86]. Moreover, carotid intima media thickness and epicardial adipose tissue thickness were found to be increased [87].

## 4. The Right Heart and the Pulmonary Artery

### 4.1. Right Ventricle

#### 4.1.1. Under Healthy Circumstances

The right ventricle (RV) is a triangular-shaped heart cavity when viewed from the sides and crescent-shaped in cross-section, and its diameter is gradually increasing from the apex to the base. The RV curves over the LV on the right side. The RV fills from the RA during diastole via the tricuspid valve (TV) and empties to the pulmonary artery via the pulmonary valve (PV) during systole. The myocardium is more trabecularized and thinner in the RV compared to the LV, resulting in a lower muscle mass (RV muscle mass is only one-fifth to one-sixth of the LV). The normal sequence of right ventricular activation begins with the contraction of the inlet and ends with the contraction of the infundibulum. The RV is composed of muscle fibers located deep in the wall that are responsible for the longitudinal movement of the RV from base to apex, during which the RV axis is shortened, and the TV moves in the apical direction and superficially located circumferential muscle fibers that are parallel to the TV fibers and are responsible for movement towards the cavity of RV (“bellows” effect). The superficial muscle fibers of the RV are connected to those of the LV, connecting the two ventricles. Consequently, the RV contracts when the LV is contracting. It is important to emphasize that the rotation and twisting motions seen in the case of the LV do not play an essential role in the RV. Heart rate, the Frank–Starling mechanism, and the autonomic nervous system are the factors regulating the RV [88,89,90].

#### 4.1.2. In Acromegaly

RV enlargement and systolic dysfunction could be detected in 4.3% and 2.2% of acromegaly patients, respectively [34]. Early studies emphasized the biventricular form of hypertrophy in acromegaly [91,92]. Increased interventricular septum, RV free wall thickness, basal and longitudinal diameters, end-diastolic and end-systolic areas, E/E’ ratio, isovolumetric relaxation time, and RV ejection time could be detected in acromegaly with reduced E/A ratio and E’ velocity. hGH and IGF-1 positively correlated with RV longitudinal diameter and indexed RV end-diastolic area. Patients with active acromegaly had increased RV index of myocardial performance, isovolumetric contraction time, and shortened RV ejection time compared to patients in remission [93]. In another study, increased deceleration time and decreased E/A were found [94]. In contrast, no significant changes in RV size, and RV free wall thickness could be detected between acromegaly patients and healthy volunteers [6].

### 4.2. Right Atrium

#### 4.2.1. Under Healthy Circumstances

**Figure 4:**
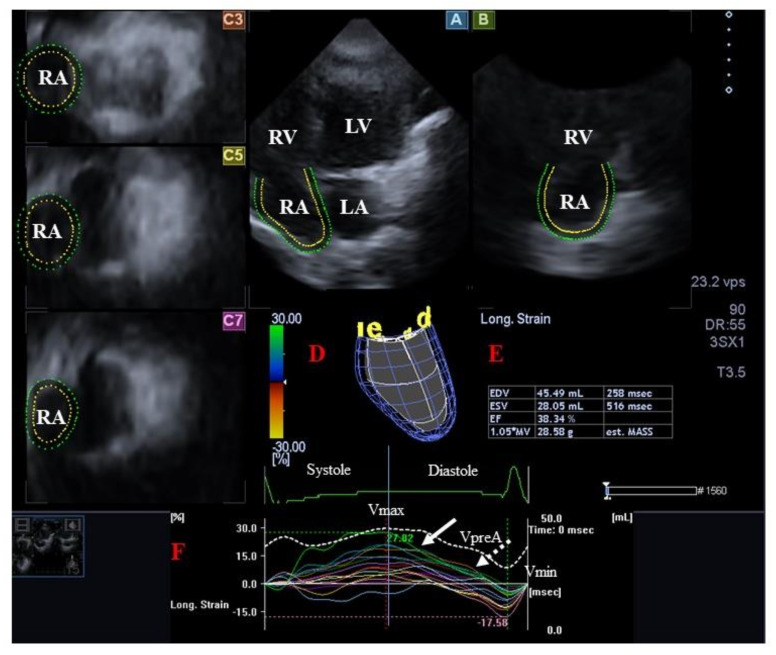
Three-dimensional (3D) speckle-tracking echocardiography-derived evaluation of the right atrium (RA). From the acquired 3D echocardiographic dataset, the following typical views are presented: longitudinal apical four-chamber (**A**) and two-chamber (**B**) views and short-axis views at basal (**C3**) and midatrial (**C5**) and superior (**C7**) RA levels. The 3D RA model (**D**) is seen together with calculated RA volumetric data (**E**) and time–RA global (white curve) and segmental (colored curves) longitudinal (**F**) strain curves with time–RA volume changes curve (dashed white curve). White arrow represents peak RA strain, while white dashed arrow represents RA strain at atrial contraction. Abbreviations. LV = left ventricle, LA = left atrium, RV = right ventricle, RA = right atrium, EDV = end-diastolic volume, ESV = end-systolic volume, EF = ejection fraction, MASS = RA muscle mass, Vmax = minimum end-systolic RA volume, VpreA = early diastolic RA volume before atrial contraction, Vmin = end-diastolic minimum RA volume.

The walls of RA are partly similar in structure to LA, having circumferential and longitudinal muscular bundles, which are larger than those in the LA. The terminal crest and terminal pectinate muscles are the main muscles that make up the wall of RA. The terminal crest is arranged longitudinally, while pectinated muscles are connected to the muscles of the atrioventricular vestibule. The rim of the oval fossa plays an equally important role for both atria [53,54]. The RA fills from caval veins and coronary sinus during systole like a reservoir (maximum RA volume), with the opening of the tricuspid valve (TV) in early diastole, RV passively fills without active atrial activity (“conduit” phase, pre-atrial contraction RA volume), followed by active LA contraction at the end of diastole after the P-wave (“booster pump” function, minimum RA volume. Not only the sinus node is located in the RA, but in accordance with its tension, it secretes atrial natriuretic peptides as well, and baroreceptors are also located in its wall [90,95] (Figure 4).

#### 4.2.2. In Acromegaly

Increased RA minor diameter could be detected in acromegaly, which correlated with hGH and IGF-1 levels [93]. Profound RA remodeling could be demonstrated, including RA dilation in all phases of RA function without changes in any RA-SV and RA-EmF values, especially in acromegaly patients with active disease in the MAGYAR-Path Study. Strain analysis revealed that RA contractility represented as RA-3DS may be enhanced in acromegaly patients with a reduction in RA strain in circumferential and longitudinal directions. In the case of inactive disease, RA-LS was similar to that of matched controls. Correlation analysis revealed that numerous independent RA strain parameters had significant correlations with different hormonal variables in active acromegaly patients. These correlations are not present in inactive acromegaly patients. These results could suggest that RA remodeling and functional changes may subside with proper acromegaly treatment [14]. Both inter- and RA intra-AEMD were found to be prolonged in acromegaly patients, with more pronounced effects in the case of active disease [58]. 

### 4.3. Tricuspid Valve

#### 4.3.1. Under Healthy Circumstances

Similar to the MV, the TV has a complex, saddle-shaped asymmetrical ellipsoid structure with a dynamic nature respecting the cardiac cycle. When the TV opens in diastole, blood flows from the RA to the RV in one direction, and the TV closes in systole without tricuspid regurgitation. The TV consists of the following components: fibrous tricuspid annulus, anterior, posterior, and septal leaflets, tendineal chords, and papillary muscles [90,96] (Figure 3).

#### 4.3.2. In Acromegaly

Tricuspid regurgitation is not a typical feature of acromegaly, and the ratio of different grades of TR is similar to that of controls [64].

### 4.4. Pulmonary Valve

#### 4.4.1. Under Healthy Circumstances

The semilunar pulmonary valve (PV) has a significant role in regulating one-way blood flow toward the pulmonary circulation. The PV opens in ventricular systole and closes in ventricular diastole [90,97].

#### 4.4.2. In Acromegaly

No clinical study demonstrating detailed acromegaly-related special abnormalities of the pulmonary valve was found in the literature.

### 4.5. Pulmonary Artery

#### 4.5.1. Under Healthy Circumstances

The pulmonary artery is one of the large arteries that carries blood from the RV to the pulmonary circulation [90].

#### 4.5.2. In Acromegaly

The diameter of not only the aorta but that of the main pulmonary artery was increased in acromegaly, and these changes were related to disease duration instead of serum hGH and IGF-1 level [68].

## 5. Pathophysiologic Background

There are a wealth of knowledge about pathophysiological abnormalities associated with acromegaly. Shortly, both hGH and IGF-1 are vascular growth factors stimulating collagen deposition. Moreover, elevated hormone levels result in increased IGF-1 receptor activation in cardiac myocytes, leading to increased mRNA expression of sarcomeric proteins, including myosin light chain-2 and troponin, responsible for increased cardiac contractility, hypertrophy, and fibrosis [98]. Direct or indirect effects of overproduction of hGH are considered to cause valvular regurgitations via increased gene expression of matrix metalloproteinases, resulting in abnormal matrix regulation. Although proinflammatory cytokine levels are also elevated in active acromegaly, inflammation, and oxidative stress do not seem to contribute to the development of atherosclerosis. Over hormonal changes, myocardial, valvular, and vascular abnormalities could further deteriorate each other, like stiffening of a large artery, which may have important implications on cardiac morphology and performance in acromegaly and may increase the susceptibility to atheromatous disease. It could be theorized that RV/LV remodeling may affect RA/LA volumes and functions and further valvular abnormalities. For instance, increased LA volumes and LA-SV abnormalities might be due to decreased diastolic LV filling. However, the effects of coexisting risk factors, like hypertension, DM, dyslipidemia, and mitral regurgitation, cannot be ruled out in the remodeling of the cardiac chambers. All these abnormalities together are responsible for changes in myocardial mechanics and vascular remodeling detailed above [64,98,99,100].

## 6. Clinical Implications

Today, patients with acromegaly are discovered early to receive treatment that prevents late-stage acromegalic cardiomyopathy. These facts may explain why old studies have shown more severe results. Moreover, advanced cardiovascular imaging techniques can help to identify abnormalities early in treated patients with acromegaly. Findings from the literature detailed above can help to detect expected alterations.

## 7. Conclusions

Acromegaly is associated with abnormalities in cardiac mechanics and vascular remodeling. Some of these alterations are related to the activity and/or the duration of the disease. Currently, due to modern treatment procedures, most acromegaly patients no longer show any signs of end-stage “classic” acromegalic cardiomyopathy, only slight cardiovascular abnormalities.

## Data Availability

Not applicable.
